# Detection of parechovirus A in respiratory, gastrointestinal, and neurological clinical samples of pediatric patients from Panama (2014–2015)

**DOI:** 10.1186/s12985-023-02268-9

**Published:** 2023-12-19

**Authors:** Lizette Gutierrez, Viridiana Sáenz, Danilo Franco, Brechla Moreno, Ediner Fuentes-Campos, Zeuz Capitan-Barrios, Luis Felipe Rivera, Jean-Paul Carrera, Juan Castillo, Marlene Castillo, Juan Miguel Pascale, Sandra López-Vergès, Néstor Sosa, Leyda Ábrego

**Affiliations:** 1 The University of Texas at El Paso (UTEP), El Paso, TX 79968 US; 2 Department of Research in Virology and Biotechnology, Gorgas Memorial Institute of Health Studies, Panama City, Panama; 3 Facultad de Ciencias Naturales, Exactas y Tecnología, Departamento de Microbiología y Parasitología, Universidad de Panamá, Panamá, Panama; 4 Carson Centre for Research in Environment and Emerging Infectious Diseases, La Peñita, Darien, Panama; 5 Department Research in Genomic and Proteomic, Gorgas Memorial Institute of Health Studies, Panama City, Panama; 6 Division of Infectious Diseases, University of New Mexico Hospital, Albuquerque, NM US

**Keywords:** PeV, PeV-A, Panama, Gastrointestinal infection, Respiratory infection, Neurological infection

## Abstract

Parechovirus A (PeV-A, Parechovirus, *Picornaviridae*) are human pathogens associated with mild to severe gastrointestinal and respiratory diseases in young children. While several studies have investigated the association of PeV-A with human disease, little is known about its epidemiology or detection in Latin America. Between the years 2014 and 2015, a total of 200 samples were collected from Panamanian pediatric patients aged < 16 years old exhibiting symptoms associated with respiratory (n = 64), gastrointestinal (n = 68), or neurological (n = 68) diseases. These samples were gathered from patients who had previously received negative diagnoses for the main respiratory viruses, rotavirus, and neurological viruses like herpes virus, enterovirus, and cytomegalovirus. The presence of PeV-A was analyzed by real time RT-PCR.

Eight positive PeV-A infections (4.0%, 95% CI: 1.7 to 7.7) were detected: two in respiratory samples (3.0%, 95% CI: 0.3 to 10.8), five in gastrointestinal samples (7.3%, 95% CI: 2.4 to 16.3), and one in cerebrospinal fluid (1.5%, 95% CI: 1.4 to 7.9). The study provides evidence of PeV-A circulation in Panama and the data collectively, remarked on the importance of considering PeV-A in the Panamanian pediatric diagnostic landscape, especially when conventional testing for more common viruses yields negative results.

## Impact statement

The presence of Parechovirus A (PeV-A) in Latin America has been an unexplored area of research, although it has been associated with gastrointestinal and respiratory diseases in young children. The study was conducted between 2014 and 2015. Two hundred samples were collected from pediatric patients with symptoms related to respiratory, gastrointestinal, or neurological illnesses, with a negative diagnosis of common viruses.

The findings revealed the presence of PeV-A in Panama with varying prevalence among the sample types: two in respiratory samples, five in fecal samples, and one in cerebrospinal fluid.

Understanding the epidemiology and detection of PeV-A in Latin America can lead to better diagnosis and management of pediatric diseases and enhance public health efforts in this region.

## Introduction

Parechovirus A (HPe-A; Parechovirus, *Picornaviridae*) are non-enveloped, single-stranded, positive-sense RNA viruses with icosahedral capsids [21]. The first strain was identified in 1956 [22]. Parechoviruses have been divided into six species, of which Parechovirus A (formerly named human parechovirus HpeV) is the only one infecting humans [20]. Other species are zoonotic and important in veterinary medicine: Parechovirus B (formerly named the Ljungan virus)(Lindberg & Johansson, [9], Parechovirus C (Sebokele virus) [6], Parechovirus D (ferret parechovirus) [19], Parechovirus E (falcon parechovirus) [14], Parechovirus F (gecko parechovirus) [17]. PeV-A has a genome of approximately 7,000 nucleotides that encode structural proteins, non-structural enzymatic genes, and conserved untranslated regions [5].

The clinical manifestations of PeV-A range from asymptomatic to severe gastrointestinal or respiratory disease in children, which can be associated with long-term neurodevelopmental sequelae. Encephalitis, meningitis, myocarditis, and sepsis have also been associated with PeV-A infection, and evidence suggests that disease severity depends on the genotype, PEV-A being subdivided to date into 19 genotypes, and the age of patients [4]. Transmission mechanisms of PeV-A occurs through fecal–oral or respiratory routes [21].

Although PeV-A cases have been reported worldwide, most of these cases are concentrated in studies undertaken in Europe, Asia, and North America [3]. Evidence of PeV-A circulation in the Latin American region is limited to a few studies on retrospective surveillance, clinical reports, and analysis of urban streams in Chile, Argentina, and Ecuador [4]. Clinical suspicion of PeV-A circulation in Panama has been suggested to be linked to febrile diseases in neonates; however, laboratory confirmation of these cases is not available [11]. Here, we aimed to detect the circulation of PeV-A in Panama by analyzing clinical samples collected during 2014 and 2015 from pediatric patients with gastrointestinal, neurologic or acute respiratory infections.

## Materials and methods

The study protocol was approved by the Gorgas Memorial Institute of Health Studies (ICGES) Bioethics Committee (no. 981/CBI/ICGES/16). From January 2014 to December 2015, nasopharyngeal (NP) and oropharyngeal (OP) swabs, cerebrospinal fluid (CSF), fecal, serum, and ocular samples were collected from pediatric patients aged < 16 years who presented with gastroenteritis, respiratory, and/or neurologic symptoms from different regions from Panama and were sent in cold chain (4^0^C) to the ICGES in Panama City, Panama. Samples were initially screened for Enteroviruses, Cytomegalovirus (CMV), Varicella-zoster virus (VZV), Herpes simplex virus (HSV), Rotavirus, Adenovirus, Influenza A, Influenza B, Human Metapneumovirus, Parainfluenza 1, 2, and 3, Rhinovirus, and Respiratory Syncytial Virus (RSV). After the first screening, only the negative samples from 200 patients were used to investigate the presence of PeV-A infections.

Viral RNA was extracted using the QIAamp® Viral Mini Kit (Qiagen, Valencia, CA, USA). Samples were tested to detect PeV-A viral RNA using a specific single-target qualitative Real Time RT-PCR with the primers ParechoF31 (5’ CTGGGGCCAAAAGCCA-3’) and K30 (5’-GGTACCTTCTGGGCATCCTTC-3’), and the probe FAM-HPeV-MGB (5’-AACACTAGTTGTA(A/T)GGCCC-3’) described by [2] with AgPath-ID ™ One-Step RT-PCR Reagents (Applied Biosystems Life Technologies) and the Applied Biosystems® 7500 Fast Real-Time PCR System. Any sample with a CT < 37 was considered positive. A sequenced-confimed positive sample was used as control.

Results are presented as proportions with bimonial 95% confidence intervals.

## Results

Of the 200 clinical samples, 68 neurological samples (CSF and serum) (34%), 68 gastrointestinal samples (fecal and eyes/mouth/anus swaps) (34%), and 64 respiratory samples (nasopharyngeal swab) (32%) were utilized for the study. Eight positive PeV-A infections (4.0%, 95% CI: 1.7 to 7.7; *n* = 8/200) were detected: two in respiratory samples (3.0%, 95% CI: 0.3 to 10.8; *n* = 2/64), five in fecal samples (7.3%, 95% CI: 2.4 to 16.3; *n* = 5/68), and one in cerebrospinal fluid (1.5%, 95% CI: 1.4 to 7.9; n = 1/68) (Table 1). Of these eight positive samples, 3 (three) were from females, 3 (three) from male and 2 (two) were of unidentified sex.

**Table 1:** Characteristics of PeV-A-positive pediatric cases in Panama, 2014–2015

Year	Age	Sex	Type of sample	Symptom	Clinical diagnosis	Observations – Initial laboratory results
2014	7 m	M	Fecal	diarrhea, vomiting, abdominal pain	Rotavirus	Negative for rotavirus
2015	1 day	F	CSF	suspected encephalitis	Herpes Virus 1 and 2	Mother with suspected Herpes Virus Encephalitis; Negative
2015	2 m	M	NP	cough, rhinorrea, respiratory distress	Respiratory syncytial virus	Negative for: Adenovirus, Influenza A, Influenza B, Human Metapneumovirus, Parainfluenza 1,2,3, Rhinovirus, Respiratory Syncytial Virus
2015	1 m	F	EMAS	pneumonia, vomiting, diarrhea	Enterovirus	Negative for enterovirus
2015	12 m	M	NP	Fever, runny nose, cough, bronchopneumonia	Unknown	Negative for Adenovirus, Influenza A, Influenza B, Human Metapneumovirus, Parainfluenza 1,2,3, Rhinovirus, Respiratory Syncytial Virus
2015	48 m	F	Fecal	diarrhea, vomiting, abdominal pain	Rotavirus	Negative for rotavirus
2015	UNK	UNK	Fecal	diarrhea, vomiting, abdominal pain	Rotavirus	Negative for rotavirus
2015	UNK	UNK	Fecal	diarrhea, vomiting, abdominal pain	Rotavirus	Negative for rotavirus

**Abbreviations**: m, months; F, feminine; M, masculine; CSF, Cerebrospinal fluid; NP, nasopharyngeal swab; EMAS, eyes, mouth and anus swab; UNK, Unknown

**Fig. 1:**
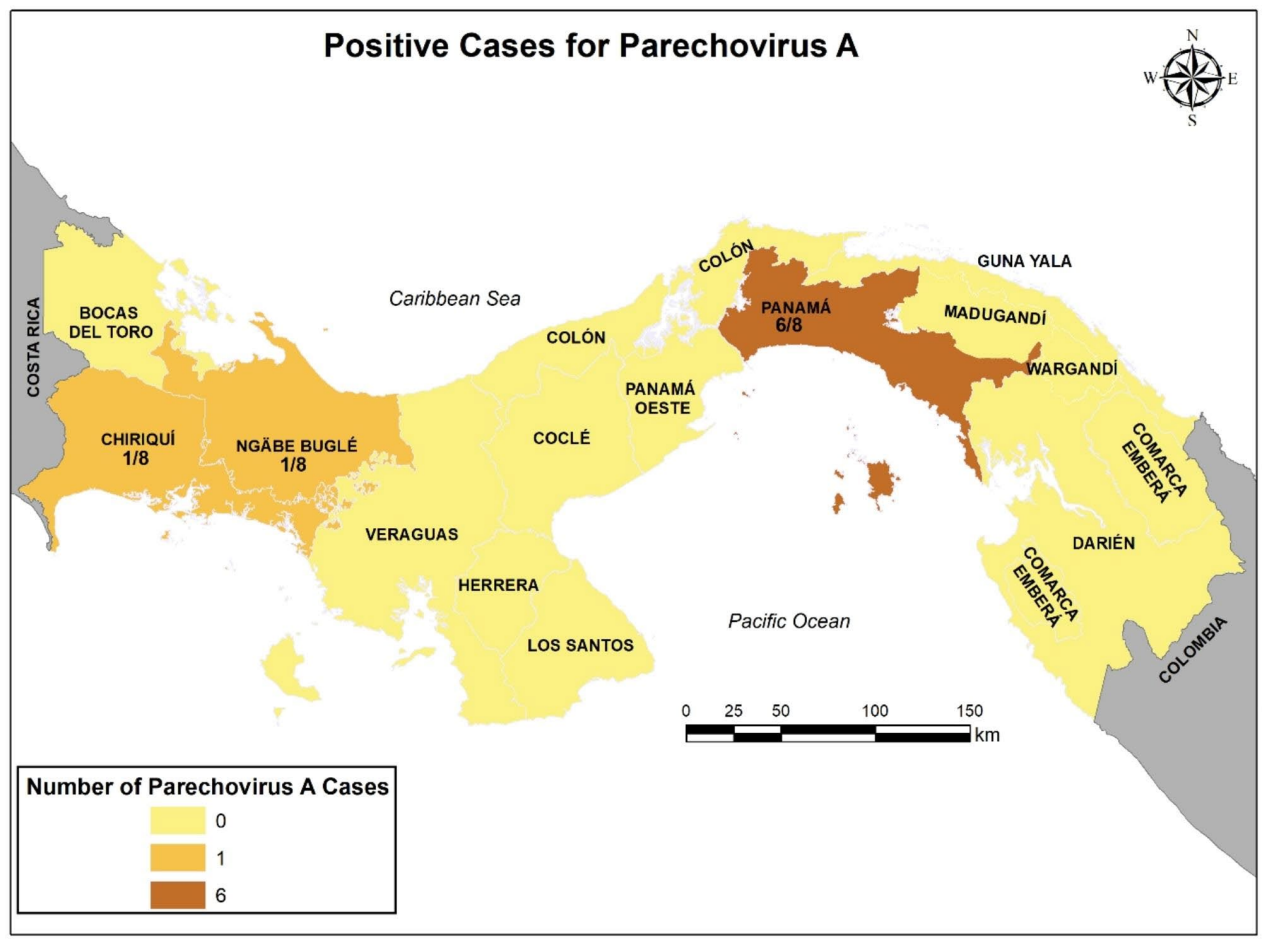
Geographical distribution of PeV-A-positive samples collected between 2014–2015 in the Republic of Panama

The analyzed samples came from all over the country. The largest number of cases came from the Hospital del Niño Dr. Jose Renan Esquivel in Panama City (128, 64%), which is the main tertiary pediatric public hospital serving patients from throughout the country (Table 1). Over 50% of the samples came from children living in the metropolitan area of the province of Panama, whereas only 1 positive sample was detected in Chiriqui, East Panama and Ngobe-Bugle native reserve (Fig. 1).

The two PeV-A-confirmed respiratory cases had rhinorrhea, cough, and respiratory distress, which began on the day of the initial evaluation. The five confirmed cases of gastrointestinal disease presented with vomiting and diarrhea, one of them also had pneumonia, and the one neurological case had encephalitis (Table 1). Other clinical symptoms included abdominal pain (50%, n = 4/8 positive cases, 80%, n = 4/5 gastrointestinal samples) and fever and respiratory distress to a lesser extent (12.5%, n = 1/8 positive cases).

A total of 119 patients (59.5%, n = 119/200) were younger than two years of age with a median age of 5 months. Of these, 5 were positive for PeV-A (4.2%, n = 5/119 of children under 2 years), all of them being less than one year old (Table 1). The 3 positive cases that were not part of this age group corresponded to gastrointestinal samples. Two of these positive patient samples had no age information, and the other positive case came from a patient of 4 years old (3.7%, N = 3/81) (Table 1).

## Discussion

While clinical suspicions have hinted at the circulation of Parechovirus A (PeV-A) in Panama [11], this study explicates compelling laboratory evidence substantiation for the actual presence of PeV-A in the country. Our findings showed that PeV-A is associated with acute gastrointestinal, respiratory, and neurological pediatric infections. Moreover, a notable trend emerged wherein the highest incidence of positive PeV-A cases was observed in gastrointestinal samples, exhibiting a frequency of 4%. This aligns with the previously documented prevalence range from 2 to 16.3% of PeV-A positivity among children presenting with diarrhea [1, 23]. This consistency lends additional weight to the findings in terms of PeV-A’s potential association with gastrointestinal manifestations [1]. This proportion of 4% was similar to that in a Nigerian study of children with a similar age range [13], but lower than that reported in studies from Chile, Spain, and Malawi [3]. Although a higher prevalence of PeV-A was obtained from fecal samples, the clinical symptoms of acute infection could not be directly related to PeV-A, as it has been shown that the duration of the virus in fecal samples can last several months [7].

The second most common symptoms in positive PeV-A samples were respiratory, with a frequency of 3%, similarly to a study carried out in China (3.43%) from 2009 to 2015, and a 10 years follow-up study (2006 to 2016) from Norway, which reported a positivity of 8.8% [18, 24]This follow-up study described that patients positive with PeV-A had mainly low respiratory tract infections [18] similarly, our positive patients reported higher and lower respiratory tract infections.

In this study, we described a PeV-A confirmed case of CNS infection in a newborn in Panama, which is consistent with previous studies showing that CNS infections are detected mainly in newborns and infants under 6 months of age [4, 10].

PeV-A infections were more common in infants under one year of age, regardless of sex, and these had more severe symptoms, these observations are similar to the results obtained in Missouri in 2019 [16]. The observed detection rate among children under 2 years of age during the analyzed years (2014–2015) stood at 2.5%. In contrast, results from Germany displayed rates exceeding 10% [1].

Our study had some limitations. A general caveat is the potential introduction of bias in the epidemiological surveillance, arised from the limited number of clinically suspected samples received in reference laboratories, primarily from hospitalized patients rather than from the outpatient setting. Another caveat, is that, even if we observed that most positive cases are infants and young children, we did not have enough samples for statistical correlation between age and severity [8] future studies with a higher number of samples encompassing more years are needed. Our monoinfection model did not encompass an assessment of coinfection, an aspect that warrants attention in future investigations due to the noteworthy coinfection rates recorded in Japan. There, 59.2% of PeV-A-positive samples exhibited coinfection with other enteric viruses [15]. It is important to note that our results do not imply the absence of co-infection within Panama. Finally, this study did not genotype the confirmed PeV-A cases, which is an important information for ensuring better medical care and general epidemiological surveillance. Indeed, some genotypes have been associated with specific types of symptoms and severity; the PeV-A1 genotype has been more frequently associated with gastrointestinal and respiratory diseases, whereas the PeV-A3 genotype is the main genotype causing CNS and severe diseases in newborns and infants [4, 10, 12] There is a need for future development in the detection and genotyping capabilities within Panama and Central America.

In summary, our findings highlight the detectability of PeV-A in acute samples collected from pediatric hospitalized patients who were initially classified as having associated infections with known etiological agents. This underscores the challenges encountered in executing a differential diagnosis when symptom profiles exhibit substantial overlap with other viral infections, including but not limited to Herpes Virus, Enterovirus, RSV, and rotavirus. In addtion, this study underscores the need for continued surveillance and research to elucidate the full scope of PeV-A’s impact on human health, specially in children, in the Latin American context.

## Data Availability

N/A
